# Defense of the brain time view

**DOI:** 10.3389/fpsyg.2015.01350

**Published:** 2015-09-04

**Authors:** Valtteri Arstila

**Affiliations:** ^1^Department of Behavioral Sciences and Philosophy, University of TurkuTurku, Finland; ^2^Turku Brain and Mind Center, University of TurkuTurku, Finland

**Keywords:** brain time view, time-marker view, perceptual asynchrony, temporal judgment, reaction time

## Introduction

Making sense of our ever-changing surroundings requires the extraction of temporal information from a continuous stream of stimulation. How do we achieve this when the relevant information can be separated by only tens of milliseconds? I address this question by focusing on the debate concerning the perceptual asynchrony of changes of visual features. This refers to the finding that when a moving stimulus simultaneously changes its color and direction of movement, the subjects report that the change in color occurs 60–100 ms before the change in direction.

One explanation for this finding is that colors are processed faster than motion, which in turn means that changes in color are processed faster than changes in motion, and this difference in perceptual latency is reflected in our judgments of the temporal features of the stimuli (Moutoussis and Zeki, 1997, 2002). Crucially, this explanation assumes that the judged order of events mirrors the time at which the brain generates the representation of the events or their features. Because the temporal properties of the representations generated by the brain serve as time-markers^1^, this view has been called *the brain time view*.

Nishida and Johnston (2002, 2010) object to this explanation and propose *the time-marker view* as an alternative. This view differs from the brain time view in two respects. First, it holds that representations of color and motion are generated at the same time, and that the reported asynchrony between them results from an error in a specialized mechanism responsible for temporal judgments. Second, temporal judgments mirror the timing of external events as closely as possible (rather than the time when the neural processing of the events is completed). For this reason, the mechanism is thought to be a mid-level perceptual process.

In what follows, I will defend the brain time view from the objections raised against it by Nishida and Johnston. This has been already done on the empirical grounds (e.g., Arnold, 2010; Moutoussis, 2012, 2014). My argumentation complements this debate by focusing on the more theoretical issues and highlighting implicit assumptions in Nishida and Johnston's argumentation.

## The inherent problems of the brain time view

The first set of objections concerns a number of inherent problems that the brain time view allegedly faces. To begin with, referring to Dennett and Kinsbourne (1992), Johnston and Nishida (2001, R428) argue that the brain time view faces “some thorny philosophical problems.” Yet, not all of them are particularly pressing. For example, Nishida and Johnston (2002) argue that the brain time view comprises “a logical pitfall” because it equates the time when the event appears to occur with the time when the brain generates the representation. Even though it is theoretically possible that these two can be separated, it does not follow that they actually are. Thus, pointing out the possibility is not a particularly effective objection.

Two inherent problems need to be addressed in more detail, however. First, Nishida and Johnston (2010, 286) claim that the brain time view suffers the “logical shortcoming of identifying physical co-occurrence of the cortical representation of event A and that of event B with the representation of co-occurrence of events A and B.” Second, in relation to the brain time view, they (Nishida and Johnston, 2002, 359) claim that “[i]n order to judge the temporal order of two arbitrary neural events, the brain must have a mechanism to compare them and anatomical connections of high temporal fidelity between the neurons to be compared. This meta-analysis of neural processing places a high combinatorial burden on the brain.” These two claims are inconsistent though. While the first holds that the co-occurrence of two neural events is not separately represented, the second argues that the temporal judgments are due to some sort of comparison mechanism. The second claim is a more plausible option and concurs with, say, Efron's (1963) idea of simultaneity center according to which the temporal order of stimuli is determined on the basis of the relative arrival times of sensory signals to this hypothetical simultaneity center. Thus, the objection based on a logical shortcoming is misdirected.

As for the claim concerning a burden on the brain, it too is misdirected as it assumes that the purpose of the brain time view is to deduce the actual time of external events from the time of neural activity—this is the reason why the temporal comparator supposedly needs to account for the temporal features of different anatomical connections. However, such an assumption is not subscribed to by proponents of the brain time view, nor is it part of the brain time view as Nishida and Johnston themselves describe it! On the contrary, the brain time view holds that timing mirrors when the representations of events are generated. (Mirroring not mean that timing needs to match exactly with the time when the representations of events are generated.) Thus, the mechanism needs to compare only the temporal properties of neural signals—just as Efron's simultaneity center does—and, since this task is necessitated by the time-marker view as well, the burden on the brain is the same in this respect.

It is worth highlighting another commonality between these two views: in both theories, time-marker is *a temporal property of neural activity brought about by an external event that some timing mechanism makes use of* ^2^. For example, in the time-marker view, such a mechanism makes use of “the temporal pattern of the neural activity elicited by [external] events.” (Nishida and Johnston, 2002, 366) Thus, the difference here is that in the time-marker view the time-marker is based on an unconscious, mid-level perceptual neural activity, whereas in the brain time view the neural activity relates to the representation of the event, and the temporal mechanism comes into play later in the processing hierarchy.

## The brain time view and inconsistent latency estimations

The second objection against the brain time view is based on inconsistencies in perceptual latency estimations obtained using the reaction time method and temporal judgment tasks^3^. Nishida and Johnston (2002, 362) claim that the inconsistent results are problematic for the brain time view because, in the context of the perceptual asynchrony debate, “it is difficult to understand why [the asynchrony measured with temporal judgments] is not reflected in reaction time.”

The described inconsistency assumes, however, that an external event produces only one time-marker and that assumption has been rejected in two equally reasonable ways. Sternberg and Knoll's *different time-marker hypothesis* (1973) rejects the assumption by maintaining that the two tasks have different task demands: temporal order judgments maximize correct judgments, whereas reaction time tasks emphasize speed (Sternberg and Knoll, 1973). Miller and Schwarz (2006, 394) likewise maintain that these two tasks have “fundamentally different task demands.” Thus, the tasks use different features of a single internal response as time-markers^4^ and subsequently produce different results based on the same response. This concurs with the previous notion of time-markers because the constitution of a time-marker depends on the timing mechanisms and, hence, one internal response can manifest as different time-markers if different timing mechanisms make use of different temporal properties of the response.

Another way to reject the assumption is to argue that an external event causes two different internal responses, both of which serve as time-markers (Tappe et al., 1994; Aschersleben and Müsseler, 1999). One response is utilized by temporal order judgments and occurs in the later stage of processing. Early on, the processing leading to this response is separated from the processing which feeds into the motor system and is used in the reaction time tasks. Because, there are two timing mechanisms that use different internal responses as the basis for time-markers, the two mechanisms can provide inconsistent results. In both alternatives, temporal judgments make use of temporal properties of neural states, which can be representations of events, and thus the brain time view can account for the inconsistency.

## Latencies of different types of changes

Finally, Nishida and Johnston (2002, 2010) make a distinction between first-order and second-order changes. The first-order changes, called *transitions*, require that two points in time be compared (e.g., color at t_1_ and t_2_). Second-order changes, called *turning points*, require the comparing of three points in time (e.g., spatial position at t_1_, t_2_, and t_3_). A special mechanism is thought to exist only for temporal judgments related to transitions and thus determining the time of turning points takes longer. Consequently, even if a transition and a turning point were to occur simultaneously, the latter would be judged to occur later than the former. Nishida and Johnston's results as regards synchrony between different transitions and turning points support this claim.

Assuming that these experiments are comparable to those concerning the original finding, the obtained results conflict with the claim that colors are processed faster than motion. However, they are not in conflict with the brain time view in general. This is because the results do not specify the processing stage at which the time-markers for the turning points are established. Hence, they are also compatible with the claim that the mechanism responsible for temporal judgments in the brain time view requires more time to determine turning points than to determine transitions. In this way, the brain time view can explain Nishida and Johnston's finding in largely the same fashion as the time-marker view.

## Summary

The brain time view and the time-marker view can be understood to rely upon the existence of a temporal judgment comparator that makes use of the temporal properties of neural activity caused by an external event. The main difference in these two views concerns the processing stage in which such comparison takes place and what the timing concerns about. The objections raised by Nishida and Johnston against the brain time view cannot settle the question of which theory is closer to the truth. However, Nishida and Johnston (2010, 286) are correct in their claim that the brain time view “assumes that a brain time mechanism is poorly designed in the sense that processing delay is added to event time estimation.” Then again, given the evidence that cortical processing influences temporal judgments (Arnold and Wilcock, 2007), and that Efron (1963) postulated his simultaneity center exactly because it could account for the processing delays between cortical hemispheres, the existence of such a poor mechanism could be closer to the truth than the mechanism postulated by the time-marker view.

## Conflict of interest statement

The author declares that the research was conducted in the absence of any commercial or financial relationships that could be construed as a potential conflict of interest.

## Footnotes

^1^In its most general sense, a time-marker is something that a process can utilize during a task in which the temporal features of stimuli or experiences of stimuli are determined.

^2^This assumes that the outcome of the mid-level mechanism responsible for temporal judgments in the time-marker view is something that we become conscious of, rather than something that is utilized by some subsequent timing mechanism. Theories that make use of this kind notion of time-markers often concern simultaneity perception and reaction time studies (e.g., Efron, 1963; Jaśkowski, 1996; Jaśkowski et al., 2014; Yarrow and Arnold, 2015). This notion can be contrasted with a symbolic notion that is often attributed to Dennett and Kinsbourne (1992). They illustrated it using date stamps on letters—a stamp (time-marker) represents the date when a letter is sent regardless of when the stamp is interpreted (when the letter arrives). This notion is rarely explicitly endorsed—something along these lines has been presented in relation to the postdiction effects (e.g., Eagleman and Sejnowski, 2000, 2007; Grush, 2005, 2006)—and even more rarely explicated. Thus, the notion remains under-described, both theoretically and in neural terms (e.g., Arnold, 2010; Arstila, 2015).

^3^For example, reaction time measurements and temporal order judgments are affected differently by changes in stimulus intensity and its luminance profile (Roufs, 1974; Jaśkowski, 1996), stimulus modality (Rutschmann and Link, 1964; Jaśkowski et al., 1990), spatial frequency of visual gratings (Tappe et al., 1994), and stimulus motion (Aschersleben and Müsseler, 1999).

^4^According to Sternberg and Knoll, TOJ tasks use the time of the activation peak of the response and RT tasks use the time when the activation crosses some earlier threshold. Miller and Schwarz, in turn, argue that the criterion in RT tasks is higher than in TOJ tasks, and thus that the RT tasks employ the later part of the internal response than TOJ tasks.
